# Nurses’ Requirements for Relief and Casualty Support in Disasters: A Qualitative Study

**DOI:** 10.17795/nmsjournal9939

**Published:** 2014-04-17

**Authors:** Mahmoud Nekooei Moghaddam, Sara Saeed, Narges Khanjani, Mansour Arab

**Affiliations:** 1 Department of Health Services Management, Kerman University of Health Sciences, Kerman, IR Iran; 2 Department of Epidemiology and Biostatistics, Kerman University of Health Sciences, Kerman, IR Iran; 3 Monash Centre for Occupational and Environmental Health, School of Public Health and Preventive Medicine, Monash University, Melbourne, Australia; 4 Department of Medical and Surgical Nursing, Kerman University of Health Sciences, Kerman, IR Iran

**Keywords:** Disaster Planning, Mass Casualty Incidents, Nurses, Qualitative Research, Relief Work

## Abstract

**Background:**

Nurses are among the most important groups engaged in casualty support, regardless of the cause, and they are one of the largest care groups involved in disasters. Consequently, these workers should gain proper support and skills to enable effective, timely, responsible and ethical emergency responses.

**Objectives:**

In this study, we investigated the needs of nurses for proper casualty support in disasters, to facilitate better planning for disaster management.

**Materials and Methods:**

This was a qualitative content analysis study. Interviews were performed with 23 nurses, at educational hospitals and the Faculty of Nursing at Kerman Medical University, who had a minimum of five years working experience and assisted in an earthquake disaster. Intensity and snowball sampling were performed. Data was collected through semi-structured interviews. Interviews were transcribed and coded into main themes and subthemes.

**Results:**

Four major themes emerged from the data; 1) psychological support, 2) appropriate clinical skills education, 3) appropriate disaster management, supervision and programming, and 4) the establishment of ready for action groups and emergency sites. The participants’ comments highlighted the necessity of training nurses for special skills including emotion management, triage and crush syndrome, and to support nurses' families, provide security, and act according to predefined programs in disasters.

**Conclusions:**

There are a wide range of requirements for disaster aid. Proper aid worker selection, frequent and continuous administration of workshops and drills, and cooperation and alignment of different governmental and private organizations are among the suggested initiatives.

## 1. Background

The history of earthquakes in Iran, and Kerman Province in particular, is well known. Statistics show that Iran is the tenth disaster prone country in the world and the fourth in Asia (1). The World Health Organization defined disaster as a situation in which people’s normal means of support for life with dignity have failed as a result of either a natural or human-made catastrophe (2). Ironically, a disaster may happen during anyone’s lifetime, and preparation is the best way to prevent adverse consequences. 

Kerman is one of the most vulnerable provinces in Iran located in the southeast of the country. Statistics show that one of the main potential hazards in the province is earthquakes and their aftermaths. Experts stated that there are eighteen dangerous fault lines in this province, and over the past 100 years, there have been more than 14 major earthquakes in this province (3). The devastating Bam earthquake, occurred on the 26th of December 2003, at 5:26:26 am local time, at a strength of 6.8 on the Richter scale. At that time, Bam population was more than 100 000 people. More than 25 000 died, 50 000 were injured, and 100 000 people lost their houses (4). Implementing proper disaster preparedness, either natural or human‐made on this scale is difficult. 

The first step for emergency support preparedness is to find out; who are the right people to engage in a relief operation, and then to determine how and what tasks they should perform. There are a number of researches, in both Iran and other countries, declaring that nurses have a very important role in all disasters, and it is essential for nurses to be prepared and trained to respond to disaster incidents (5-9). Nurses are the largest group of health‐care workers, they lie at the forefront of medical care and would therefore play a key role in any major disaster relief operation (7-9). All health care responders, including nurses, must have knowledge of the key principles of disaster medicine (5). Jorgensen et al. believed that nurses’ lack of knowledge and understanding of disaster science and hazards framework, may hinder proper management of disaster events (6). Without education and training, nurses may not be prepared to function effectively in a disaster relief, especially in a manner that is productive, efficient, collaborative, and minimizes stress (9). In disaster relief operations, preparation is important, but readiness for the unexpected and quick reaction to changes are crucial (10). Therefore, like other countries, recognizing nurses’ needs in a disaster and responding to them quickly is very important, and this research aimed to recognize those needs, especially in earthquakes.

## 2. Objectives

There are many experienced nurses working in Kerman, with valuable experience concerning aid and casualty support after earthquakes. For this reason and to deeply and comprehensively understand the needs of nurses for aid and casualty support, we designed a qualitative study to help us further in understanding, constructing and applying the proper educational and technical programs for disaster management. We expect the results of this study to be invaluable for other developing countries as well.

## 3. Materials and Methods

We conducted a qualitative, content analysis study. The participants were nurses working at Kerman Medical University (KMU) educational hospitals (i.e. Afzalipoor Hospital, Shafa Hospital, and Bahonar Hospital), or the KMU Faculty of Nursing, who had more than five years’ experience in general nursing practice. Participants where sought through intensity and snowball sampling. The first information rich participants were selected from nurses intensely involved in casualty care and who had provided assistance in previous disasters such as the Bam earthquake; and the next participants were introduced to the researchers by the previous participants. 

Interviewees were approached in person and invited to participate in the study. Sampling was continued until data saturation. Open semi-structured individual interviews were used for the data collection. Initially a broad question was asked about what the nurse saw and did in a disaster, then more focused questions were asked about the requirements that the nurse thought should have been in place to provide better aid to the casualties. If needed, the interviewer would prompt the interviewee by asking him or her to explain further, for example; did they think they had enough theoretical knowledge, was there a need for more demonstrations and workshops, and if they felt confidence enough to participate in another rescue in the future. After the interview the participants were provided with the researcher’s phone number, and it was possible for them to add other things after their interview if they remembered anything else important. 

Before starting, the protocol was approved by Kerman Medical University Standing Ethics Committee. The study topic, aim and method of interviewing were explained to the participating nurses, and written consent was obtained. A single researcher performed all the interviews, and her accompanying assistant took care of the recording and hand notes. All interviews were recorded, kept as audio files and transcribed word by word on the same day. Twenty three nurses were interviewed, and each interview took between one to two hours. The interview time and date was organized according to each nurse’s preference. After typing the interviews, any ambiguity in the text was cleared by contacting the participants and asking for further explanation. 

### 3.1. Data Analysis

Data collection and analysis were performed simultaneously. Each interview was read in depth several times for familiarization. Codes and categories were extracted by an inductive process through line by line reading of the text. Consensus about the coding was achieved among the authors. Furthermore, two people who had no role in the data collection read, commented and confirmed the coding. After completing the coding and assuring its accuracy, the main concepts were identified and extracted. All four researchers participated in the data analysis. 

According to Guba and Lincoln, credibility, dependability, confirmability and transferability, describe rigor in qualitative research (11). Credibility was achieved by member checking. We took the final report to the participants and asked if they felt the results were a true and accurate reflection of their words. Their comments and statements were used to improve the results. Dependability was assessed by peer check strategies. Confirmability was documented by leaving an audit trail and asking a colleague to follow the path and comment on the conclusions. The study was transferable as the results were sensible and logical for nurses other than the ones participated in the study. 

## 4. Results

Questions about preparedness for casualty support in disasters, reminded most of our interviewees about the Bam earthquake. Many participants believed that the experience gained from this earthquake could play an important role for disaster management in the future. 

Fortunately, in this qualitative study we had the chance to document some of these experiences and discuss them. Four major themes emerged from the data.

### 4.1. Theme I: Psychological and Mental Support

The most frequent topic in the collected data was mental and psychological stress which experienced by both traumatized people and caregivers. The anxiety of some caregivers was so intense that in many cases affected their performance. “Some of our colleagues, proportionate to the amount of mental pressure, could not perform well. Nurses who had years of experience could not work properly. They could not even do a simple task!” Some participants thought that the nurses who had worked in emergency departments or in the war zone (during the Iran-Iraq war), those with more experience, and those who were practically and routinely involved in the care of trauma patients, had less anxiety and were calmer in dealing with the casualties. Some of our participants even suggested that counselors should be available at the scene to provide mental and psychological support. “Nurses working in the aftermath of a disaster should have frequent psychological counseling, and after a shift someone has to sit down and talk to them, to make sure they are not facing (psychological) problems; are neglected and no one has anything to do with them, if nurses have psychological problems themselves, they cannot support the injured.” Many participating nurses confessed that they did not know the proper way of dealing with their patients’ stress. They believed that they should know how to deal with people who are anxious, violent, physically and verbally abusive and people who shout, scream or do not calm down easily.

### 4.2. Theme II: Clinical Educations Needs

The participating nurses frequently mentioned the necessity of learning about trauma complications and trauma care (Box 1). They listed some of the important instruments which should be available in post-disaster care, and aid personnel should be expert or very familiar with their practice (Box 2). All nurses believed that they should use protective equipment to prevent the transmission of communicable diseases, and to learn and apply safety measures when necessary. Nevertheless, after the Bam earthquake, some of the aid personnel were so busy attending to the casualties forgetting their own safety. “In the Bam disaster we were under severe mental stres, no one paid attention to things, such as wearing gloves or masks. Nobody used protective equipment properly.”

We found out from most of the interviewees that due to their busy schedules and lack of time, nurses themselves were not particularly active in learning on their own. They also mentioned the fact that there was no surveillance to oblige them to use and practice the latest evidence. “Most of us nurses have reached a scientific standstill. We do not practice according to the new scientific evidence. I usually use my experience instead.”

There was a lot of interview text about the necessity of conducting drills and the characteristics of an efficient drill. We have summarized the information in Box 3. Some participants commented that the drill schedule should be easy to understand and not complicated, and there should be external evaluators who assess nurses’ gain out of every drill. Some participants believed that a good drill should be very close to the real situation and that they should not announce the day of the drill in advance, it should be announced suddenly. “After the Bam earthquake several drills were held. We were informed in advanced. The problems we faced with (real) casualties were different from what was in the drill. We did not have the stress we had in the (real) post-disaster situation during the drill. Drills are never like the (real) disaster. People who participate in the drill know that it is fake.”

Some of the nurses thought that the flow charts programmed for disasters are not efficient and what is said is not what is performed. "All of the personnel here (in this hospital) had been through (post-disaster aid) training and had attended the drills, but after the Bam earthquake all of them were confused and did not know what to do. It was quite interesting to see that those who were at the drills and had been through the trainings were less present in the actual disaster scene and had trouble with the practical parts.”

Some participants thought that aid workers should be given a specific code that determines their duty or scope of work at the time of disaster aid and this code should be consistent, clear and determined in advance. Training should be planned properly according to their duties. There should be an organized schedule to perform accordingly in a disaster.

### 4.3. Theme III: Scene Management, Supervision and Programming

All of the nurses referred to the crucial role of strong, well trained management in disaster control. They said that after the Bam disaster, the crowds of volunteers who rushed to offer help caused a lot of chaos, and along with weak management led to the misuse of human resources. Sadly, the aid givers were not organized and had not been given identity cards in advance. “Our knowledge is of no use, there should be order!” Almost all of the nurses believed that in disasters there has to be a clear aid and rescue program and the proper amount of equipment and facilities should be prepared and stored in advanced. Moreover, they suggested that there should be a particular storage site for disaster aid equipment in hospitals, which in the case of disasters could be accessed promptly, without bureaucracy and time wasting. One nurse commented that he knows that in the hospital he is working at that there is such a storage unit, but it is only for 40 to 50 casualties, which is not enough. More than a half of the participants expressed their concern over the lack of sufficient equipment or inaccessibility to this equipment after disasters. All of the nurses believed that triage was important. But, some of them commented that it is very difficult or even impossible to perform this when there is no order. The interviews showed that strong management and surveillance over triage and patient classification is essential (Box 1). According to the nurses, the Bam earthquake led to the planning of a more organized disaster control chart, but they did not know how effective it would be in the next disaster.

### 4.4. Theme IV: Ready for Action Aid Teams and Sites

Several participants suggested that there should be certain teams made up of aid workers and trained volunteers with strong personalities, interested, energetic and physically fit. The selected personnel should be trained intensively and frequently in both theoretical and practical aspects, they should be ready for action and familiar with up-to-date skills. They also commented that ordinary people and untrained volunteers should not be allowed to enter the circle of aid workers; because they cause various problems. “There were many people who did not help and even prevented others from working. They were crowding and causing chaos everywhere. They wanted to help, but they were actually causing trouble for the personnel work.” Most nurses wanted clear planning and their duties to be specified in advance to prevent confusion in the actual event. They were also worried about the shortage of nurses, and said that they would face many problems, if another disaster such as the Bam earthquake occurs.

The nurses’ comments also raised the important issue of allocating predetermined places for admitting the injured. Planning in advance could allocate big public halls or stadiums for admitting the injured. Also, each hospital should announce a limit for patient admissions in disasters according to its availability of extra spaces, equipment and personnel. “Hospitals have to announce beforehand, how many patients they can accept; so that more patients are not sent there. Otherwise, patients would face problems and we would lose patients we could save.” 

Many nurses said that the disaster scene should be kept safe and under control. They insisted that the presence of police forces is essential for their safety and tranquility. Others commented that different organizations such as; the Red Crescent, police, charity organizations, social workers, municipality and the army, should be present at the scene and cooperate closely with each other to ensure safety and to prevent opportunistic actions.

They also commented that reliable groups should take responsibility for supporting the nurses’ family, so that they can work without worry. Eventually, many nurses confessed that they were not prepared for another earthquake or disaster. Some of the comments about preparedness for the next earthquake are listed in Box 4.

**Box 1.** Nurses’ Educational Needs for Casualty Support According to the Experience and Comments of the Participants

Educational Essentials for Disaster Aid Nurses	Suggested Teaching and Support Methods for Each Skill
**Staying calm, self-control, avoiding anxiety in dealing with the injured; Psychologically supporting the injured and their families; Preparedness in dealing with nervous, desperate and violent people**	Removing nurses who undergo too much stress or psychological pain; Education about self-control, alleviating anxiety and stress; Assigning work periodically in emergency rooms or with severely injured patients; Presence of social workers, psychologists and support groups at the scene
**Dealing with the injured; First aid; Seeking critical signs in trauma patients; Triage (priority 1, 2, 3)**	Films and videos; Assigning work periodically in emergency rooms or with severely injured patients; Continuous education workshops; Drills
**Knowledge about trauma and casualty care, including: Simple and complex fractures; Contusions; Crush injuries; Internal bleeding; Kidney damage (in crush injuries); Spinal injuries; Psychological disorders; Burns; Wound care; Interpreting ECG (the essentials); Interpreting radiographies (the essentials); Serums and proper IV therapy for special injuries (such as crush injuries); Drug interactions (especially in cases of multiple doctor visits)**	Films and videos; Continuous education workshops; Drills Pamphlets, handbooks
**Practical skills, such as: Intravenous injections (especially for children); Using urinary catheters; Wound dressings, bandages; Suturing; Bleeding control; Injections (IV, IM); Proper casualty rescue from the rubble; Proper casualty moving; Using fracture braces; CPR (single and double administered); Intubation; Using the artificial respiratory machine; Working in field hospitals**	Continuous education workshops; Drills, at least biannually
**Personal safety; Insisting and reminding that personal safety comes first**	Continuous education workshops; Drills
**Speed and accuracy; Working as a team**	Drills, at least biannually
**Leadership; Management; Order**	Drills, at least biannually; Determining duties and responsibilities in advance
**English skills; to communicate with foreign aid workers; to attend foreign workshops; to get information from the internet**	Workshops; Classes
**Proper reporting and information exchange: Through the internet; Through a predefined reporting system**	Workshops
**Legal and ethical considerations; what may/may not lead to malpractice charges; what are his or her legal obligations; what to perform/not to perform; what to learn/do not need to learn**	Workshops

**Box 2.** Essential Equipment for Front Line Disaster Aid (From Nurses’ Perspective)

Equipments
**IV set (IV stand, fluids, angiocatheter),**
**Suturing set**
**Injection set**
**Sterile dressing and bandage set, sterile gauzes**
**Necessary medicine, including: Narcotics, Analgesics, Tetanus vaccines**
**Portable monitoring devices**
**Casualty transfer equipment (wheelchairs, brancards, stretchers)**
**CPR equipment**
**Fracture braces**
**Ventilator**
**Portable sonography unit**
**Chest tube**
**Sphygmomanometer**
**Flash lights**
**Spatulas**
**Oxygen capsules**
**Gowns, masks, gloves, goggles**
**Prepared and packaged food**
**Warm [appropriate] clothes, blankets**
**Personal hygiene equipment**
**Resting facilities**

**Box 3.** Nurses’ Suggestions (in Brief) for Improving Disaster Drills

Suggestions
**Continuous, regular and up to date education**
**Capable, mentally strong and energetic nurses enrolled**
**Repetition, 2 to 6 times a year (so that each person can attend at least 1 to 2 times a year)**
**Practical and theoretical education together**
**Easy to follow and understandable materials**
**Similar to reality as much as possible**
**The role of casualties should be handed over to nursing or medical students or people who have enough knowledge to play the role correctly**
**Performed without notice and by sudden recall**
**Surveyed and monitored constantly**
**A special committee assigned for surveillance and monitoring drills**
**The drill should be accounted as one duty shift for the nurse**
**Nurses’ lack of time and heavy workloads should be taken care of**
**A code/responsibility should be assigned to people in advance**
**The role or responsibility should be consistent during the different drills**
**Substitutes should be assigned**
**A fixed, well-rehearsed program to practice during the drills and to apply in disasters**
**Managers determined in advance and well-rehearsed in their role**

**Box 4.** Summary of Comments for Better Disaster Aid in the Future

Comments
**Managers, supervisors, coordinators determined in advance**
**Aid teams with pre-determined members, these people should have: Extensive theoretical and practical training; Their substitutes determined and ready to act in case of their absence**
**Aid teams should include: Nurses; Surgeons, general and orthopedics; Technicians, casualty transfer; Social workers; Cleaners; Security or police**
**Transfer facilities should be foreseen in advance and ready to go: Ambulances, helicopters, trucks**
**Casualty accommodation foreseen and the maximum capacity of each site determined**
**Field hospital ready to establish**
**Necessary medical equipment stored in advance**
**Medical equipment kept at a specific site, ready to use (without bureaucracy)**
**Equipment routinely checked for expiry date and substituted**
**Aid workers’ rest and work times well programmed, using substitute aid workers properly**
**Volunteers well managed and taken care of**
**Police or security forces ready to act and enter the scene immediately**
**Social workers present to support grieving families and orphans**
**Cleaners working continuously for hygiene and disinfection**
**An active information service in charge of reporting the situation and registering casualty information**
**Patient information safe and accessible**
**Acknowledgment of the hardworking staff after the event**

## 5. Discussion

The participants’ experience showed the importance of the nurses’ educational and non-educational needs for aid and casualty support in disasters, especially in developing countries with weak infrastructures. Some of these needs have been confirmed in other studies. O'Boyle et al. mentioned that nurses in disaster situations may have to work in a chaotic situation with scarce resources and inefficient management for casualty care, and they may also experience high levels of mental and psychological stress (12). Other studies have concluded that education and preparedness of nurses is necessary to decrease emotional and psychological stress (12-14), and that nurses have to know how to reduce their own stress (2). Chapman et al. reported that stress control for caregivers is one of the most important issues should be taught in disaster workshops. They should also have access to consultation services (15). Due to the broad range of psychological and mental problems mentioned after disasters, we suggest that one of the main educational topics for disaster caregivers should be how to overcome personal stress and anxiety and how to deal effectively with these types of stresses in others.

Both in our study and in previous ones, researchers have concluded that effective education and planning for disasters can increase nurse’s confidence, knowledge and clinical skills (15). It has also been reported that knowing how to protect oneself and provide immediate care for the injured, recognizing their own role and limitations, and knowing where to seek additional information and resources is necessary for nurses’ involved with disaster aid (16, 17). We understood from the nurses’ interviews that continuous and frequent theoretical education has been beneficial. Others studies have also shown that continuous education could affect nursing practices effectively (18), and that disaster aid education can decrease mortality rates, improve health indices, and decrease disaster expenses (2). In a study by Shadel et al. participants were in favor of 30 to 60 minutes sessions and suggested methods of learning such as video records, train-the trainer, and paper-based self-learning packages (19). 

In this study, some nurses commented that nurses are frustrated and were not motivated to learn new materials due to their heavy workloads and lack of time to participate in educational programs. In addition, O’Boyle et al. noted that nurses’ hesitancy to participate in disaster education or drills may be due to; time constraints, financial limitations, and competing demands (12). Weiner et al. showed that nursing school programs provide limited curricula for disaster preparedness, and 75% of respondents thought that nurses were inadequately prepared in disaster management (20). 

In our study, many nurses openly confessed that they were not prepared for the next disaster. The nurses in our study commented that they needed assurance that their families would be supported or taken care of during their leave. Similarly, French et al. mentioned nurses’ needs and the conflict they experience between family and work commitments, particularly when they are simultaneously disaster victims and caregivers. In response to Hurricane Floyd, nurses preferred being with their families and experienced increased anxiety when separated from their family members. Some even lost their jobs when they decided to stay with their families (21). Furthermore, in another study, nurses expressed concern over their personal safety, their family’s safety, family commitments, insufficient food, water and resting facilities (12). O’Boyle et al. asserted that the inclusion of care for nurses’ families may be an effective way to support nurses who work in disasters. Developing systems for childcare and elderly care during emergencies could reduce the burden of competing responsibilities for nurses (12). 

In our study, all participants insisted that sufficient equipment and facilities should be prepared in advance and according to a schedule. O’Boyle et al. also stated that nurses are concerned about the lack of equipment in disasters, because many hospitals operate with a “just-in-time” system, even though hospitals are supposed to be ready to supply additional materials and equipment during a crisis (12). All of the nurses in our study agreed that there was a need for strong management and organized planning to prevent chaos and provide a suitable working environment. 

Other studies have also mentioned that one of the important items that should be taught in disaster education programs is crowd control and hospital security (15). In another study, nurses expressed concern about working in a chaotic environment after a disaster. They predictedthat lack of leadership, unclear chain of command, lack of role clarity, unpreparedness, and difficulty in coping, would result in such a situation. There were also concerns about an unsafe clinical environment and loss of freedom to leave the hospital due to inadequate staff (14). In our study nurses also insisted that strong management is needed to coordinatee and organizee working and rest shifts of nurses, organizee replacements, and the efficient use of human resources. In other studies, the importance of availability of relief staff is highlighted, so that nurses are able to take breaks, and recruitment of additional staff from other hospitals, have also been mentioned (12). In addition, the need for previously prepared practical protocols, and the need for qualified and real team work in the situation, have been mentioned in another study (7). 

Among the other comments made by our participants was the need to preselect certain places to accommodate casualties and to determine the maximum capacity of each site based on its characteristics. Chapman et al. have also mentioned that determining the capacity of each hospital and the number of patients each one can handle, should be performed in advance (15). 

In our study, many participants thought that experienced nurses who had previously worked with severely injured people, such as in emergency wards and ICU nurses, were able to work more efficiently in the disaster scene. A competency report about nurses responding to mass casualty incidence stated that assigned roles of professional nurses in a mass casualty incidence may vary based on their diverse educational backgrounds, experiences, and practice settings, within the community and health care system (16). It is believed that although nurses world-wide must have minimum levels of knowledge and skill to appropriately respond to a mass casualty incident, not all nurses can or should be prepared as first responders (16). 

Similar to our results in Box 3, Gebbie et al. commented that emergency preparedness and response plans have to be practiced regularly and understood properly (22). The Joint Commission on the Accreditation of Healthcare Organizations in the US requires regular emergency management drills at least once and in some states twice a year. They believe that drills allow nurses to practice performing their roles, to give feedback on each other’s performances, and to improve disaster aid plans (22). Others have stated that performing drills for disaster aid is an effective way to examine personnel’s preparedness for a real disaster (23), especially when performed with casualtyy simulation moulage and smart simulated victims (15).
